# Molecular genetic characterization of myeloid neoplasms with idic(X)(q13) and i(X)(q10)

**DOI:** 10.3389/fonc.2024.1428984

**Published:** 2024-09-26

**Authors:** Marta Brunetti, Kristin Andersen, Gunhild Trøen, Francesca Micci, Signe Spetalen, Andrea Lenartova, Maren Randi Tandsæther, Ioannis Panagopoulos

**Affiliations:** ^1^ Section for Cancer Cytogenetics, Institute for Cancer Genetics and Informatics, The Norwegian Radium Hospital, Oslo University Hospital, Oslo, Norway; ^2^ Department of Pathology, The Norwegian Radium Hospital, Oslo University Hospital, Oslo, Norway; ^3^ Institute of Clinical Medicine, Faculty of Medicine, University of Oslo, Oslo, Norway; ^4^ Department of Haematology, Oslo University Hospital, Rikshospitalet, Oslo, Norway

**Keywords:** acute myeloid leukemia, myelodysplastic syndrome, cytogenetics, idic(X)(q13), i(X)(q10), array comparative genomic hybridization, pathogenic variants

## Abstract

**Background/Aim:**

Isodicentric [idic(X)(q13)] and isochromosome [i(X)(q10)] are infrequent aberrations in neoplastic diseases. The former is mainly reported in elderly women with myelodysplastic syndrome (MDS) and acute myeloid leukemia (AML), whereas the latter is mostly found as a secondary aberration or part of complex karyotypes in various types of neoplasms, including MDS and AML. Here, we present the molecular genetics and clinical features of six patients with myeloid neoplasia and the above-mentioned aberrations.

**Patients and Methods:**

Array comparative genome hybridization (aCGH) and next-generation sequencing (NGS) myeloid panel were used to examine genetic alterations in five bone marrow samples containing neoplastic cells carrying idic(X)(q13) and one sample with i(X)(q10).

**Results:**

The breakpoints of idic(X)(q13) were clustered within a 200 kbp region encompassing *FAM236B*, *DMRTC1B*, and *DMRTC1*. The breakpoint of i(X)(q10) was identified within a 112 kbp region on sub-band p11.22 containing *SSX2*, *SSX2B*, and *SPANXN5*. Pathogenic variants of *TET2* were identified in four cases, *SF3B1* in three cases, *ASXL1* and *SRSF2* in two cases each, whereas *STAG2*, *RUNX1*, *U2AF1*, and *TP53* pathogenic variants were detected in only single cases.

**Conclusions:**

The breakpoints of idic(X)(q13) are within a 200kbp. i(X)(q10) in our study turned out to be a cryptic idic(X)(p11) aberration, reported for the first time here. *TET2*, *SF3B1*, *ASXL1*, or *SRSF2* were highly prevalent in patients with idic(X)(q13)/i(X)(q10) abnormalities and were often associated with a worse prognosis.

## Introduction

An isodicentric chromosome with breakage and reunion at band Xq13, idic(X)(q13), and an isochromosome of the long arm of the chromosome X, i(X)(q10), are rare cytogenetic abnormalities in cancer (1, 2). The last update (April 15, 2024) of the “Mitelman Database of Chromosome Aberrations and Gene Fusions in Cancer” (1) contains 47 entries carrying an idic(X)(q13) and 55 entries with i(X)(q10). idic(X)(q13) has been predominantly observed in elderly women diagnosed with either myelodysplastic syndrome (MDS) or acute myeloid leukemia (AML), often representing the sole cytogenetic aberration in most cases (1, 3–8). Conversely, i(X)(q10) was found mostly as a secondary aberration within complex karyotypes across various neoplasms, including MDS and AML (1). Single cases of AML and MDS had i(X)(q10) as the only cytogenetic abnormality (9, 10).

Detailed characterization of the genomic breakpoint in band Xq13 has been reported in only a few cases of MDS/AML (5, 11, 12). MDS/AML patients with idic(X)(q13) were also found to carry additional sub-microscopic genetic aberrations in their bone marrow cells (5, 13). No investigation on possible additional genetic aberrations has been reported in cases with i(X)(q10). The primary consequence of i(X)(q10) is considered to be the loss of Xp and the gain of several genes on Xq. Moreover, additional genetic abnormalities, including the pathogenic variants of Tet methylcytosine dioxygenase 2 (*TET2*) gene, have been associated as a common secondary event in patients with idic(X)-positive myeloid malignancies (5).

Because of the rarity of myeloid neoplasms carrying idic(X)(q13) or i(X)(q10) and the incomplete understanding of their pathogenetic mechanisms, we here present the molecular cytogenetics and characterization of pathogenic variants in five patients of myeloid neoplasms with idic(X)(q13) and one patient with i(X)(q10).

## Materials and methods

### Materials

Information about the patients’ sex, age, diagnoses, and outcomes is given in **Table 1**. All patients were women between 63 and 86 years old. Flow cytometry was performed as part of the diagnostic routine (**Table 1**). The study was approved by the Regional Committee for Medical and Health Research Ethics (REK, project number 2010/1389; http://helseforskning.etikkom.no). All patient information has been de-identified.

**Table 1:** Clinicopathological data and hematological findings of six patients with myeloid neoplasms.

Patient	Sex/Age	Diagnosis	Immunophenotype	Treatment	Outcome
1	F/79	MDS/AML	NA	None	Died for other causes/8 months after MDS diagnosis
2	F/76	MDS/AML	CD45 weak+, CD34+, CD71 weak+, CD117+, CD123+, CD38+, HLA-DR+, CD11b-, CD13+, CD14-, CD15-, CD16-, CD33-/heterogeneous weak+, CD36-, CD64-, CD7-/heterogeneous+, CD19-, CD56-, 7.1-	azacitidinex25 cytarabine +venetoclax	Alive/4 years after diagnosis
3	F/75	MDS	NA	None	Died/7 months after diagnosis
4	F/63	MDS	NA	azacitidn x2 decitabine x5 allogeneic hematopoietic stem cell transplantation	Alive/6 years after diagnosis
5	F/79	MDS	NA	None	Died for other causes/8 months after diagnosis
6	F/86	AML	CD45+, CD34+, CD 117+, HLADR+, CD16-, CD13+, CD11b-, CD 10-, CD 64-, CD 35-, IREM 2-, CD14-, CD36+(14%), CD 105+, CD33+, CD71-, CD15+(9%), NG2-, CD123+ weak, CD38+, TdT + (47%), CD56-, CD7+(55%), CD19-, cyCD3-, cyMPO-, CD79a-, CD3-	low dose of cytarabine	Died of refractory AML/12 months after diagnosis

MDS/AML, myelodysplastic syndrome/acute myeloid leukemia according to International Consensus Classification (ICC2022); F, female; NA, Non available.

### Methods

#### G-banding and karyotyping

Bone marrow cells were short-term cultured and harvested; chromosomal preparations were stained for G-banding analysis and analyzed as previously described (14). The karyotypic description followed the International System of Cytogenomic Nomenclature (15). We received 1 to 5 samples for each patient to monitor the disease.

#### DNA extraction, array comparative genomic hybridization (aCGH), and detection of gene variants

DNA was extracted from bone marrow cells using the Maxwell 16 Instrument System and Maxwell 16 Cell DNA Purification Kit (Promega, Madison, WI, USA) or by using EZ1 Advanced XL (Qiagen, Hilden, Germany). The concentration was measured with a Quantus fluorometer (Promega) or Qubit fluorometer (Thermo Fischer Scientific, Waltham, MA, USA).

aCGH was performed using CytoSure array products (Oxford Gene Technology, Begbroke, Oxfordshire, UK) following the company’s protocols. The reference DNA was Promega’s human genomic female DNA. The slides (CytoSure Cancer +SNP array) were scanned with the Agilent Sure Scan Dx microarray scanner using Agilent Feature Extraction Software (version 12.1.1.1). Data were analyzed using the CytoSure Interpret analysis software (version 4.9.40). Annotations were based on the human reference sequence GRCh37/hg19.

Gene variants were detected using next-generation sequencing (NGS) panels designed for myeloid neoplasms. For cases 1, 2, and 5, the VariantPlex Myeloid Panel, including 75 genes, was used (Archer DX, Boulder, 2477 55th St #202, United States) and sequenced on the NextSeq 2000 (Illumina, San Diego, CA, United States). For cases 3, 4, and 6, the TruSight Myeloid sequencing panel (Illumina, San Diego, CA, United States), including 54 genes, was used and sequenced on the MiSeq (Illumina, San Diego, CA, United States). Data analyses were performed following the companies’ recommended software using Archer Analysis 6.2.7 or Variant Studio3.0, respectively. Annotations were based on the human reference sequence GRCh37/hg19 and pathogenicity was determined by using relevant databases: Molecular Tumor Board Portal (MTB) Portal (Karolinska Institutet), Human Somatic Mutation Database (HSMD; Qiagen), ClinVar (National Institute of Health), Catalogue of Somatic Mutations in Cancer (COSMIC; Sanger Institute) and the World Health Organization (WHO) classification (16).

## Results

**Figure 1:**
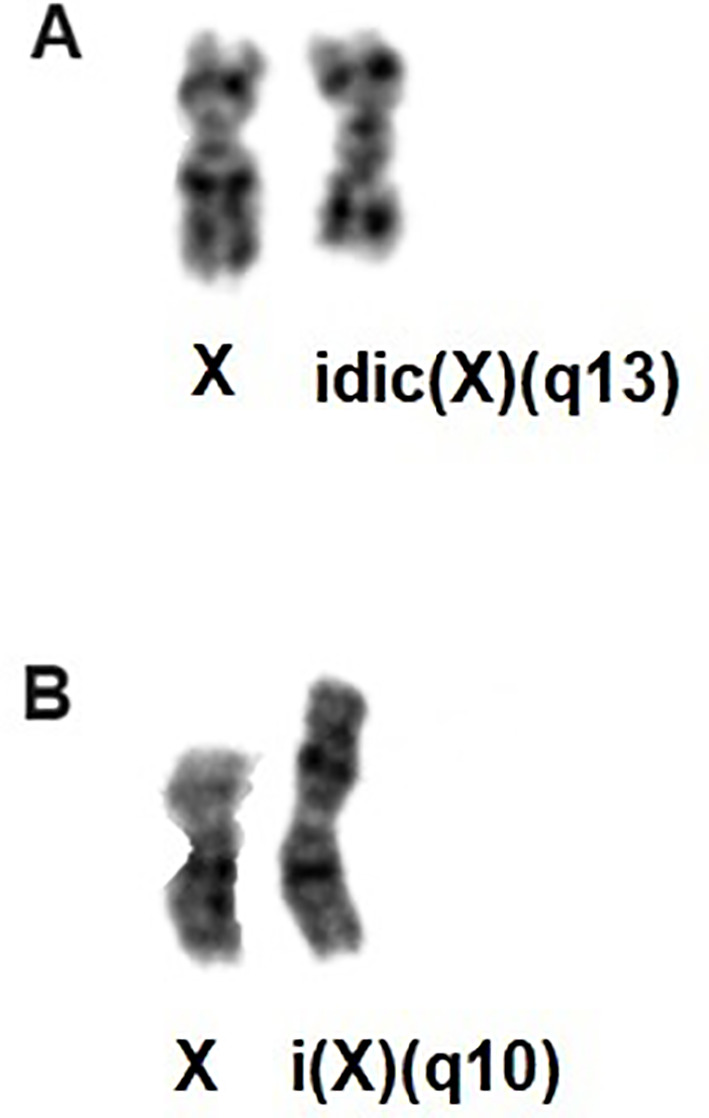
**(A)** Partial karyotype showing the idic(X)(q13); **(B)** Partial karyotype showing i(X)(q10).

The six cases were selected because their bone marrow aberrant karyotypes included an idic(X)(q13) or i(X)(q10) (**Table 2**, **Figure 1**). Cases 1 to 5 harbored one or two copies of idic(X)(q13), whereas patient 6 carried i(X)(q10) as the sole aberration (**Table 2**). **Table 2** shows karyotypic data from all samples we received.

**Table 2:** Cytogenetic and pathogenic/likely pathogenic variants detected in the six patients with myeloid neoplasms.

Patient	Sample*	Karyotype	Gene variant	Accession number**	VAF
1	Sample 1	46,X,idic(X)(q13[7]/46,XX[10]	*TET2* c.1014_1015del; p.Asn338LysfsTer12 *TET2* c.2102del; p.Gln701ArgfsTer3 *SF3B1* c.2098A>G; p.Lys700Glu	NM_001127208.2 NP_001120680.1 NM_001127208.2 NP_001120680.1 NM_012433.3 NP_036565.2	41% 40% 24%
2	Sample 1 – Day 0	46,X,idic(X)(q13)[9]/46,XX[2]	NA	NA	NA
	Sample 2 - 384 Days	46,X,idic(X)(q13)[6]/46,XX[7]	*SRSF2* c.284C>T; p.Pro95Leu *TET2* c.4133G<A; p.Cys1378Tyr *STAG2* c.1782C>G; p.Tyr594Ter *ASXL1* c.1934dup; p.Gly646TrpfsTer12 *TET2* c.4645_4669dup; p.Val1557AlafsTer29 *RUNX1* c.1008_1044dup; p.Tyr349ProfsTer263	NM_003016.4; NP_003007.2 NM_001127208.2; NP_001120680.1 NM_006603.4; NP_006594.3 NM_015338.5; NP_056153.2 NM_001127208.2; NP_001120680.1 NM_001754.4; NP_001745.2	45% 44% 44% 35% 21% 17%
3	Sample 1 – Day 0	46,X,idic(X)(q13)[5]/47,idem,+idic(X)(q13)[2]/46,XX[3]	NA	NA	NA
	Sample 2 – 282 Days	46,X,idic(X)(q13)[5]/47,idem,+idic(X)(q13)[6]/45,X,-X[2]	*TET2* c4045-1G>A *TET2* c.4600C>T; p.Gln1534Ter *TET2* c.3632G>A; p.Cys1211Tyr *SF3B1* c.2098A>G; p.Lys700Glu	NM_001127208.2 NM_001127208.2; NP_001120680.1 NM_001127208.2; NP_001120680.1 NM_012433.2; NP_036565.2	55% 42% 5% 36%
4	Sample 1 – Day 0	46,XX,der(2)add(2)(p25)del(2)(q34)[5]/46,XX[6]	NA	NA	NA
	Sample 2 – 282 Days	46,X,idic(X)(q13)[5]/46,XX[5]	NA	NA	NA
	Sample 3 - 790 Days	46~48,X,idic(X)(q13)[10],+ idic(X)(q13)[3],+1[4][cp10]/46,XX[2]	NA	NA	NA
	Sample 4 - 945 Days	46,X,idic(X)(q13)[7]/46,XX[6]	NA	NA	NA
	Sample 5 – 1106 Days	46~48,X,idic(X)(q13)[9],+idic(X)(q13)[3],+1[1][cp9]/46,XX[3]	*TET2* c.1648C>T; p.Arg550Ter *SRSF2* c.284_307del24; p.Pro95_Arg102del	NM_001127208.2; NP_001120680.1 NM_001195427.1; NP_003007.2	83% 58%
5	Sample 1	46,X,idic(X)(q13)[3]/46,XX[22]	*U2AF1* c.470A>C; p.Gln157Pro *ASXL1* c.1934dup; p.Gly646TrpfsTer12 *TP53* c.637C>T; p.Arg213Ter	NM_006758.2; NP_006749.1 NM_015338.5; NP_056153.2 NM_000546.5; NP_000537.3	41% 34% 3%
6	Sample 1	46,X,i(X)(q10)[7]/46,XX[4]	*SF3B1* c.2098A>G; p.Lys700Glu	NM_012433.2; NP_036565.2	45%

NA, Not available; *days after the diagnosis; **gene and protein accession number according GRCh37 - hg19 - Genome – Assembly; VAF, variant allele frequency.

**Figure 2:**
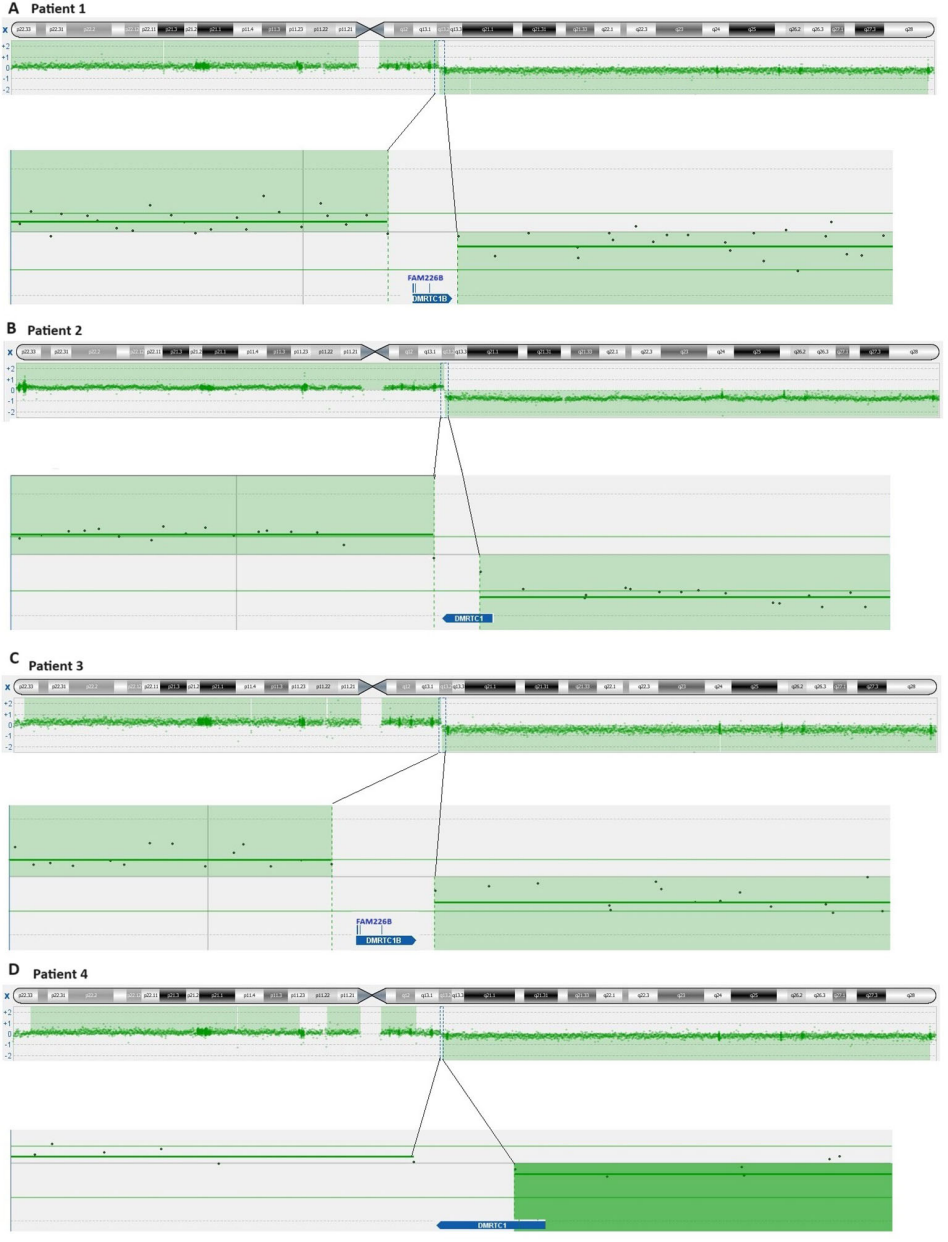
Ideogram of chromosome X and overview of genes clustering the breakpoint idic(X)(q13) according to the imbalances detected by aCGH. **(A)** Patient 1; **(B)** Patient 2; **(C)** Patient 3; **(D)** Patient 4.

**Figure 3:**
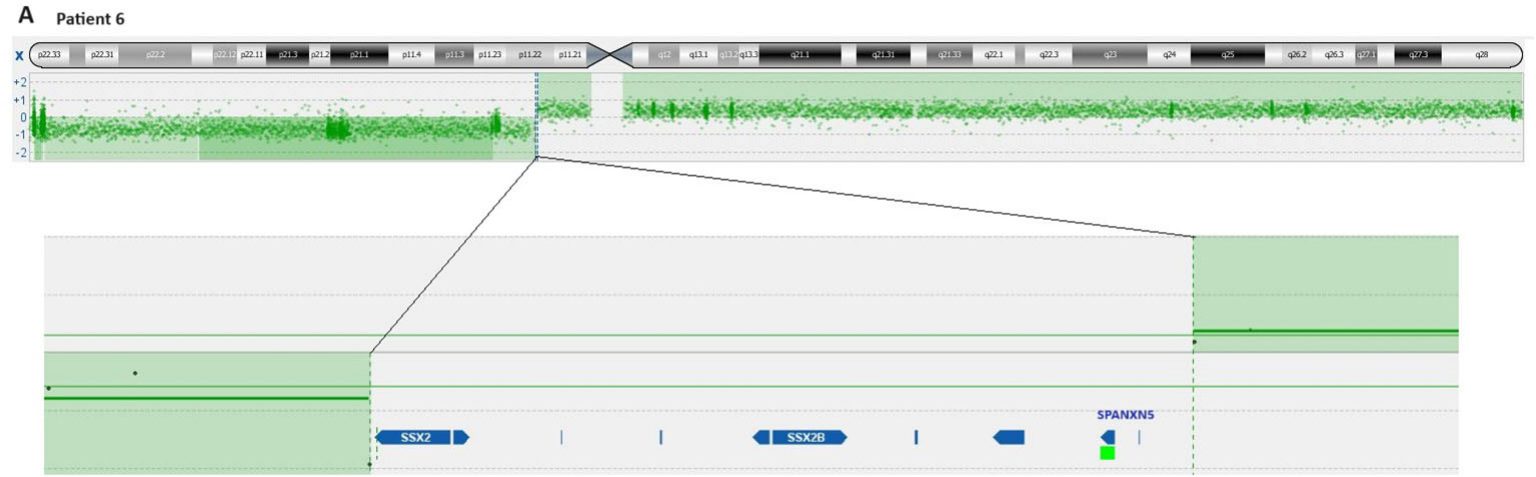
Ideogram of chromosome X according to the imbalances detected by aCGH and overview of genes clustering the cryptic breakpoint idic(X)(p11) in patient 6.

aCGH analysis revealed that in cases 1 to 4, the breakpoints of idic(X)(q13) were within a 200 kbp region on chromosomal sub-band Xq13.2, encompassing the genes Family with Sequence Similarity 236 Member B (*FAM236B*), DMRT Like Family C1B (*DMRTC1B*), and DMRT Like Family C1 (*DMRTC1*) (**Table 3**, **Figure 2**). However, aCGH did not detect any genomic imbalances in case 5, likely due to the small size of the clone carrying idic(X)(q13). In case 6, with i(X)(q10) as the sole cytogenetic abnormality, aCGH showed that the breakpoint was within a 100 kbp region on chromosome sub-band Xp11.22 (genomic position ChrX:52,725,935-52,838,474). This region includes the genes SSX Family Member 2 (*SSX2*), SSX Family Member 2B (*SSX2B*), and SPANX Family Member N5 (*SPANXN5*) (**Table 3**, **Figure 3**).

**Table 3:** The X chromosome breakpoints in the six idic(Xq)/i(Xq)-positive myeloid neoplasms by aCGH.

Case no.	Breakpoint region on the X chromosome according to GRCh37/hg19 assembly	Size	Genes
1	71,951,773-72,080,035	130 kbp	*FAM236B*, *DMRTC1B*
2	72,080,996-72,146,832	66 kbp	*DMRTC1*
3	71,951,819-72,080,383	130kbp	*FAM236B*, *DMRTC1B*
4	72,080,324-72,146,730	66 kbp	*DMRTC1*
5	No genomic imbalances		
6	52,725,935-52,838,474	112 kbp	*SSX2*, *SSX2B*, *SPANXN5*

Using a myeloid NGS panel, all six cases were found to harbor genetic variants, classified as pathogenic/likely pathogenic, in more than one gene (**Table 2**). Specifically, four patients had variants in *TET2* (cases 1-4), three had variants in *SF3B1* (cases 1, 3, and 6), and two had variants in *ASXL1* (cases 2 and 5) or *SRSF2* (cases 2 and 4) (**Table 2**). The variant allele frequency (VAF) indicated that all samples had major and minor neoplastic clones carrying the pathogenic variants (**Table 2**).

## Discussion

In our study, idic(X)(q13) was found as the sole chromosome abnormality in four out of the six patients (patients 1-3 and 5) (**Table 2**). The initial G-banding analysis of a bone marrow sample drawn from case 4 on day 0 showed a rearrangement involving chromosome 2 as the only clonal change (**Table 2**). However, upon re-evaluation of the karyotype, one metaphase was found to contain the idic(X)(q13) abnormality. Since this observation was not sufficient to confirm the aberration as clonal, the metaphase was not reported in the initial findings. In sample 5 of case 4, received at 1106 days after the diagnosis, the idic(X)(q13) abnormality was observed alongside an additional chromosome 1. Notably, this was the only instance where idic(X)(q13) was accompanied by other chromosomal aberrations (**Table 2**).

For patients 1-4, aCGH analysis detected the loss of an 83.6 Mbp region on the q arm of chromosome X, spanning from chromosome sub-band Xq13.2 to band Xq28, and the gain of a 72 Mbp region from Xp22.33 to Xq13.2. Additionally, the breakpoints on Xq13.2 were identified within the X:71,951,773–72,146,832 region. In case 5, aCGH analysis could not detect genomic imbalances, likely due to the presence of a small clone carrying idic(X)(q13) (only three out of the 25 examined metaphases had idic(X)(q13)). We compared our findings with the results published by Paulsson et al. (5), converting the genomic position from the human genome reference NCBI36/hg18 to the human reference sequence GRCh37/hg19. The region in which the breakpoints were found in the present study corresponds to the region referred to as “72.1–72.3 Mb” by Paulsson et al. (5). This region is rich in repetitive DNA sequences and contains two large segmental duplications harboring the genes *FAM236B*, two copies of *FAM236D*, and two copies of *DMRTC1* (5). Little is known about these genes. However, *DMRTC1* appears to encode a protein related to double-sex- and mab-3-related transcription factors (17, 18). It contains a region named “DMRT-like” but lacks the DNA binding domain of DMRT transcription factors (17, 18). The protein is predicted to be nuclear, part of the chromatin, and acts as a transcriptional regulator (19).

For case 6, carrying the abnormality i(X)(q10), aCGH analysis revealed, for the first time, that the breakpoints of the rearranged chromosome X occurred in sub-band Xp11.22, within the genomic region interval ChrX:52,725,935-52,838,474. Consequently, the aberrant chromosome X was not a true isochromosome but rather a cryptic idic(X)(p11). Notably, a similar finding was observed with i (17)(q10) found in many hematologic neoplasms. The apparent i (17)(q10) also had the breakpoints in the proximal part of the short arm, specifically at 17p11, indicating that the i (17)(q10) is actually a cryptic idic (17)(p11) (20).

The approximately 112 Kbp genomic region ChrX:52,725,935-52,838,474 is rich in repetitive DNA sequences, contains many segmental duplications, and harbors the genes *SSX2*, *SSX2B*, and *SPANXN5*. Breakpoints in this region have also been reported in patients with Turner syndrome carrying idic(X)(p11), and they have been linked to repetitive DNA sequences and segmental duplications. The repetitive sequence configurations at the breakpoint intervals suggest non-allelic homologous recombination as a principal mechanism for the formation of idic(X)(p11). Non-allelic homologous recombination may also be the mechanism for the formation of idic(Xq13) in myeloid neoplasms since the breakpoints occur in a region rich in repetitive DNA sequences containing two large segmental duplications (5, 21).

*SSX2* gene belongs to a multigene family of cancer-testis antigens and can be found overexpressed in multiple malignancies, e.g., in colorectal carcinoma, prostate cancer, breast cancer, hepatocellular carcinoma, glioma, lymphoma, leukemia, gastric carcinoma, and thyroid carcinoma tissue samples (22). In addition, it has been shown to be elevated in approximately one-third of acute myeloid leukemia (AML) patient samples at presentation, and it has been suggested to be a relevant target antigen for chimeric antigen receptor (CAR) therapy (23). *SSX2B*, an important paralog of *SSX2*, may function as a transcription repressor and can, together with *SSX2*, potentially be useful targets in vaccine-based immunotherapy (23). Moreover, the *SPANXN5* gene is expressed in normal testis and some melanoma cell lines, suggesting that it could also be a potential target for cancer immunotherapy (24).

In all patients, concurrent with cytogenetic aberrations, multiple pathogenic or likely pathogenic gene variants were detected using targeted sequencing (NGS-panel) (**Table 2**). The tumor suppressor gene *TET2*, located at chromosomal band 4q24, was the most commonly altered gene (four out of six patients). This finding is consistent with two previous studies, where pathogenic variants of *TET2* were frequently observed in idic(X)-positive myeloid malignancies, suggesting their involvement as early oncogenic events (5, 13). Pathogenic or likely pathogenic variants of *TET2* have been detected in a significant proportion of myeloid malignancies (5, 7, 13); they can occur in MDS (30%) and in AML (10%) (25, 26). Most of these variants lead to truncation of the TET2 protein, resulting in its reduced or lost function. The acquisition of additional pathogenic variants in other genes, such as *SF3B1, ASXL1, or SRSF2*, can influence the clinical phenotype, leading to modifications in clone morphology, type of lineage production defect, myeloproliferative features, or rates of progression to AML (27). In our study, the MDS cases, which harbored pathogenic or likely pathogenic variants of *TET2*, also carried pathogenic variants of additional genes, indicating a worse outcome. Song et al. (28) reported that double *SF3B1/TET2* pathogenic variants were most common in patients with MDS. Patients with only *TET2* variants were more likely to be diagnosed with AML than those with *SF3B1* mutations. In conclusion, our data showed that the breakpoints in the idic(X)(q13) are found in a narrow region, 200kbp, of sub-band Xq13 and that the i(X)(q10) is formally an idic(X)(p11). The breakpoint regions were rich in repetitive DNA sequences containing large segmental duplications, indicating non-allelic homologous recombination as a principal mechanism for the formation of idic(X)(q13)/idic(X)(p11). All patients showed additional pathogenic variants in more than one gene. Pathogenic variants of *TET2 g*ene were the most commonly identified, conferring a worse outcome for the patients.

## Data Availability

The original contributions presented in the study are included in the article/supplementary material. Further inquiries can be directed to the corresponding author.
